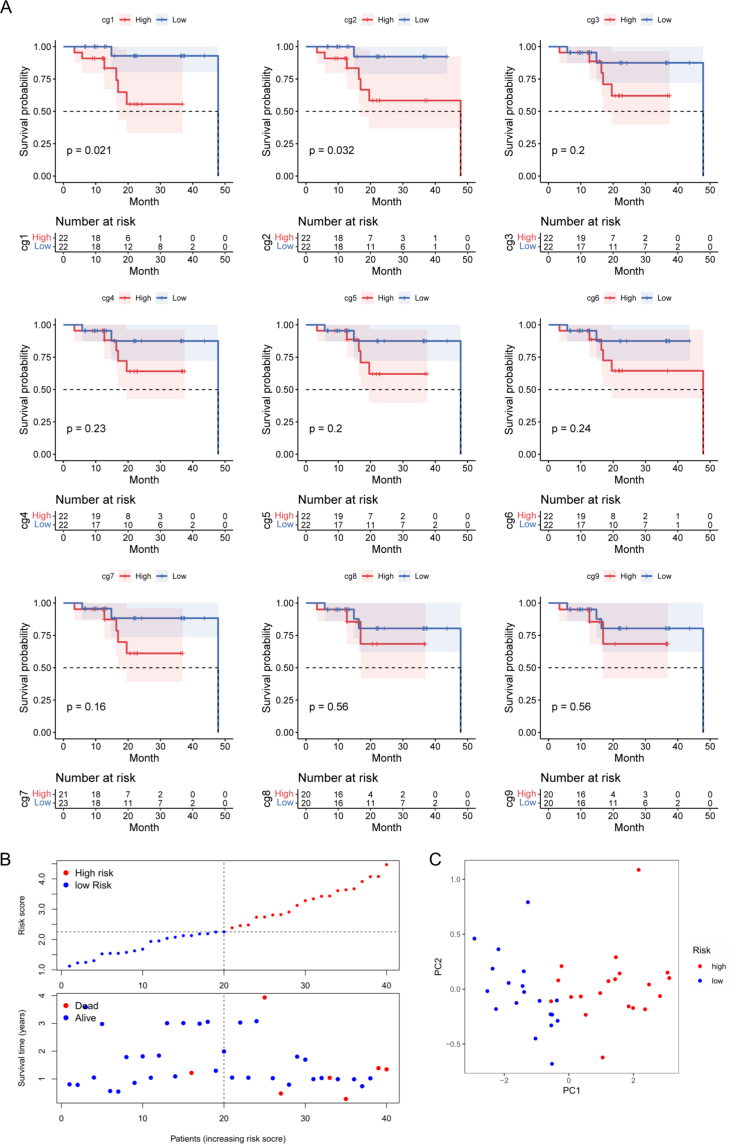# MAL2 DNA methylation serves as a biomarker for the diagnosis and prognosis of glioma

**DOI:** 10.1016/j.gendis.2023.101082

**Published:** 2023-09-07

**Authors:** Hao Luo, Xing Xiao, Weiliang Hou, Jing Cai, Ming Chen, Qisheng Tang, Yusheng Tong, Zengxin Qi, Kaicheng Li, Liang Chen

**Affiliations:** a Department of Neurosurgery, Huashan Hospital, Shanghai Medical College, State Key Laboratory of Medical Neurobiology, MOE Frontiers Center for Brain Science and Institutes of Brain Science, Fudan University, Shanghai 200040, China; b National Center for Neurological Disorders, Shanghai 200040, China; c Shanghai Key Laboratory of Brain Function and Restoration and Neural Regeneration, Shanghai 200040, China; d Tianqiao and Chrissy Chen Institute Clinical Translational Research Center, Shanghai 200040, China; e Shanghai QuietD Biotechnology Co., Ltd., Shanghai 201210, China

Glioma is a common and malignant brain tumor, and molecular diagnostics for glioma have received increasing attention.^1^^,^^2^ Previous studies have suggested that the MAL2 gene may be involved in the transcytosis of various cancers.^3^ This study aimed to investigate the potential of MAL2 as a biomarker for glioma. The candidate MAL2 CpG sites were validated by pyrosequencing and used to construct a diagnostic model for glioma. Survival analysis was also conducted to determine the relationship between highly methylated MAL2-specific CpG sites and the prognosis of glioma. The findings also showed that MAL2 was more highly methylated in glioma than in other cancers. The constructed diagnostic model can distinguish glioma from other cancers with high sensitivity (93.3%) and specificity (86.5%). Additionally, a risk score model was built based on MAL2 methylation to assess the prognosis of glioma.

MAL2 belongs to the MAL proteolipid family and is involved in transcytosis, an intracellular transport pathway used to deliver membrane-bound proteins.^4^ Although MAL2 is widely expressed in both normal and tumor tissues, it is mainly enriched in most tumor tissues. However, in both lower-grade glioma (LGG) and glioblastoma (GBM), MAL2 was significantly down-regulated (Fig. S1A). Moreover, MAL2 was significantly down-regulated in GBM than in LGG (Fig. 1A). The receiver operating characteristic curve (ROC) of The Cancer Genome Atlas (TCGA) and Genotype-Tissue Expression Project (GTEx) databases revealed that glioma could be distinguished from normal brain tissues by MAL2 expression (Fig. 1B). In the TCGA database, we selected MAL2-related genes by Pearson correlation analysis (*r* > 0.5, *P* < 0.05), which were analyzed through gene ontology (GO) and Kyoto Encyclopedia of Genes and Genomes (KEGG) enrichment analysis (Fig. S2). Taken together, these analyses indicated that MAL2-related genes were involved in synaptic-related functions and ion channel activity in glioma, and MAL2 might cause cognitive changes in glioma patients. Kaplan–Meier (KM) analysis showed that glioma patients with high expression of MAL2 tended to have longer overall survival (OS) in the TCGA database (Fig. 1C). In the CGGA mRNA-seq 693 and 325 datasets, the results were similar (Fig. S1B, C).

**Figure 1:**
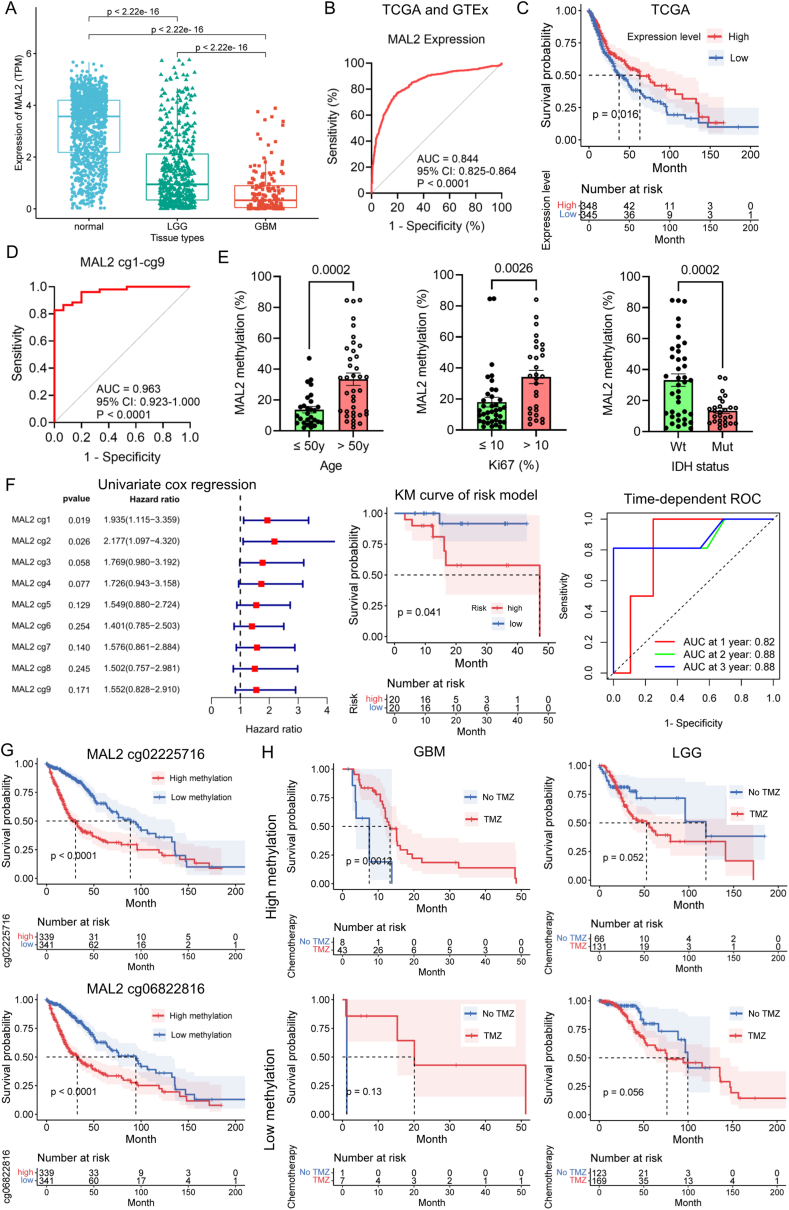
MAL2 serves as a biomarker for glioma diagnosis and prognosis evaluation. **(A)** MAL2 expression in normal and glioma tissues. The comparison of mRNA expression levels of MAL2 in normal brain tissues (*n* = 1152), LGG (*n* = 529), and GBM (*n* = 168) in TCGA indicated that MAL2 expression was dramatically down-regulated in glioma (*P* < 2.22e-16), and the expression level of MAL2 in GBM was significantly lower than that in LGG as well (*P* < 2.22e-16). **(B)** ROC curve showing the ability of MAL2 expression to discriminate between glioma and normal tissues. Combined with TCGA and GTEx database, the ROC curve of MAL2 expression was used to distinguish glioma and normal brain tissues (AUC = 0.844, 95% CI: 0.825–0.864, *P* < 0.0001). **(C)** KM analysis of MAL2 expression in glioma patients. In the TCGA database, the KM curve of MAL2 expression shows that high MAL2 expression glioma (*n* = 348) has longer survival than the low expression glioma (*n* = 345) (*P* = 0.016). **(D)** MAL2 methylation-based diagnostic model. The diagnostic model was constructed by applying multivariable logistic regression analysis and could successfully differentiate between glioma and non-glioma samples. There was a sensitivity of 93.3% and a specificity of 86.5% at the optimal cutoff value of 0.7627 (AUC = 0.9628, 95% CI: 0.9228–1.000, *P* < 0.0001). **(E)** Correlation of the MAL2 methylation status with certain clinical parameters. The correlation between the methylation levels of MAL2 and different age groups, Ki67% groups, and IDH mutation status groups: MAL2 methylation levels in different age groups: age ≤ 50 (*n* = 26), age > 50 (*n* = 36), *P* = 0.0002; the relationship between methylation levels of MAL2 and Ki67(%); two Ki67(%) groups: Ki67(%) ≤ 10 (*n* = 37), Ki67(%) > 10 (*n* = 29), *P* = 0.0026; the correlation between MAL2 methylation levels and IDH mutational status: IDH wild type (*n* = 36), IDH mutation (*n* = 26), *P* = 0.0002. **(F)** The risk score model generated based on the methylation of specific MAL2 CpG sites from Huashan samples. Through univariate Cox regression, CpG sites with *P* < 0.2 were included in LASSO Cox regression. Patients with low risk presented longer OS through KM analysis. Time-dependent ROC showed AUC at 1 year = 0.82, AUC at 2 years = 0.88, AUC at 3 years = 0.88. **(G)** KM analyses of MAL2 cg02225716 and cg06822816 methylation levels in TCGA samples. Glioma patients with low methylation at both MAL2 cg02225716 (cg1) and cg06822816 (cg7) in the TCGA database had longer OS (*P* < 0.0001). **(H)** A high MAL2 methylation level can be used to guide TMZ treatment for patients with GBM. GBM patients in the TCGA cohort with high methylation of the genes in the prognostic model had longer survival if they took TMZ [with TMZ (*n* = 43), without TMZ (*n* = 8), *P* = 0.0012]. For GBM patients with low methylation, there was no difference between the TMZ (*n* = 7) and non-TMZ (*n* = 1) groups (*P* = 0.13). For LGG patients with high methylation, there was no significant difference in the TMZ (*n* = 131) and non-TMZ (*n* = 66) groups (*P* = 0.052). For LGG patients with low methylation, there was no significant difference in the TMZ (*n* = 169) and non-TMZ (*n* = 133) groups (*P* = 0.056).

Owing to the extremely low expression of MAL2, it is challenging to define the cutoff value of the diagnostic model to distinguish glioma, as minor experimental errors might lead to a marked reduction in diagnostic accuracy.^5^ Small alterations in MAL2 expression may reflect significant differences in the methylation degree at diverse DNA CpG sites. Therefore, the methylation levels of all known MAL2 CpG sites of diverse tissues in TCGA were plotted, and MAL2 cg06822816 (cg7) was selected based on its differential methylation in gliomas versus other cancers (Fig. S3A). In addition, the other eight adjacent CpG sites, including the known cg02225716 (cg1), as well as unknown cg2–cg6 and cg8–cg9, were also further studied (Fig. S3B). Then, glioma, nonglioma tumor, and nontumor samples from Huashan Hospital were pyrosequenced for these selected MAL2 CpG sites (Fig. S3C). The methylation levels of glioma (II–IV) at the selected CpG sites were generally higher than those in other tissue samples (Fig. S3D). Next, nonglioma brain tumors and nontumor brain diseases were included in the nonglioma group, and different grades of glioma were included in the glioma group. There were clear significant differences in glioma and nonglioma samples at the nine selected MAL2 CpG sites (Fig. S4A). The ROC curves at the nine CpG sites all showed an area under the curve (AUC) > 0.80, *P* < 0.0001 (Fig. S4B). A diagnostic model considering the nine MAL2 CpG sites was built by multivariable logistic regression. The ROC curves of the integrated model showed that the predictive efficiency of the combined model was better than that of any indicator alone. Finally, the optimal cutoff value was determined to be 0.7627 with a sensitivity of 93.3% and a specificity of 86.5%, which indicated that the prediction model can distinguish glioma from nonglioma (Fig. 1D).

To describe the correlation of clinical features with the methylation level of the selected MAL2 CpG sites in glioma, the average of the methylation levels of MAL2 cg1–cg9 was used to represent the MAL2 methylation degree. Clearly, there was no difference in the average MAL2 methylation level in different groups stratified based on sex, body mass index, Karnofsky performance status, longest diameter of the tumor, volume, or P53 status (Fig. S5). In contrast, the methylation level (cg1–cg9) of younger patients was lower than that of elderly patients. Similarly, the methylation level of the Ki67 ≤ 10% group was below that of the Ki67 > 10% group, and samples with low average methylation were concentrated in IDH-mutant gliomas (Fig. 1E). This indicated that MAL2 methylation levels might exert a certain impact on the prognosis of glioma patients.

To further assess the prognostic potential of the degree of MAL2 methylation at diverse selected CpG sites, glioma patients from Huashan Hospital were followed up, and 44 participants had complete follow-up information. Due to the relatively short follow-up time and small sample size, the results of survival analysis are not all highly significant. According to KM analysis, patients with higher methylation levels at MAL2 cg1 (cg02225716) and cg2 usually had significantly shorter survival and a similar trend was also found at MAL2 cg3 to cg9. In other words, patients with high methylation levels of MAL2 had shorter survival times than those with low methylation levels, to some extent (Fig. S6A).

Next, MAL2 CpG sites (cg1–cg9) were subjected to univariate Cox regression analysis, and CpG sites with *P* < 0.2 were included in the LASSO Cox regression analysis. The constructed risk score model could be used to evaluate the prognosis of glioma patients. Patients with high risk had shorter OS than those with low risk, and time-dependent ROC curve analysis revealed the following AUC values: AUC at 1 year = 0.82, AUC at 2 years = 0.88, AUC at 3 years = 0.88 (Fig. 1F). With the increase in risk, OS was shortened, and the mortality rate increased (Fig. S6B). Additionally, the principal component analysis plot showed that patients with high or low risk could be well divided (Fig. S6C).

Then, methylation levels and clinical data in the TCGA database were applied to validate our experimental results. As expected, glioma patients with low methylation in MAL2 cg02225716 (cg1) and cg06822816 (cg7) in the TCGA database had longer OS (*P* < 0.0001) (Fig. 1G). Additionally, the value of our methylation-based model in predicting treatment response was explored in GBM and LGG patients in the TCGA database and plotted with KM curves, and the average methylation level of MAL2 cg1+cg7 was used in the following analysis. Patients were divided into high and low methylation groups according to the median methylation level of MAL2 cg1+cg7. Regarding GBM patients with high methylation, patients taking temozolomide (TMZ) tended to have longer survival. For GBM patients with low methylation, there was no difference between the TMZ and non-TMZ groups. For LGG patients, regardless of high or low methylation level, there was no significant difference between the TMZ and non-TMZ groups (Fig. 1H). Together, the findings showed that the MAL2 methylation level may serve as a therapeutic biomarker specifically for GBM.

Overall, the findings indicated that MAL2 could serve as a biomarker for glioma patient diagnosis and prognosis evaluation to improve patient outcomes. The MAL2 methylation-based diagnostic model has the potential to aid in the early diagnosis of glioma. The risk score model based on MAL2 methylation could provide valuable information for evaluating the prognosis of glioma patients. Additionally, the average methylation level at MAL2 cg02225716 and cg06822816 could guide the application of TMZ treatment, especially in GBM patients.

## Ethics declaration

The collection of samples and clinical information in this study was approved by the Ethics Committee of Huashan Hospital, and our study was approved by The Institutional Review Board of Huashan Hospital, Fudan University. Furthermore, written informed consent was obtained from each patient. All authors approved the final manuscript and its submission to this journal.

## Author contributions

Hao Luo, Zengxin Qi, Kaicheng Li, and Liang Chen designed this study. Zengxin Qi and Liang Chen recruited participants, surgical procedures, and pathology reviews. Xing Xiao, Weiliang Hou, Jing Cai, Ming Chen, Qisheng Tang, and Yusheng Tong collected and stored samples. Hao Luo, Xing Xiao, Weiliang Hou, and Zengxin Qi collected and analyzed experimental data. Hao Luo, Weiliang Hou, Xing Xiao, Zengxin Qi, Kaicheng Li, and Liang Chen wrote and edited the manuscript. All authors read and approved the final manuscript.

## Conflict of interests

All authors declare that we have no conflict of interests.

## Funding

This study was supported by grants from the 10.13039/501100001809National Natural Science Foundation of China (No. 82272116), Shanghai Municipal Science and Technology Major Project of China (No. 2018SHZDZX01) and ZJLab, 10.13039/501100003399Science and Technology Commission of Shanghai Municipality, China (No. 20Z11900100, 20S11905600), MOE Frontiers Center for Brain Science; Shanghai Shenkang, China (No. SHDC2020CR3073B), and Shanghai Zhou Liangfu Medical Development Foundation “Brain Science and Brain Diseases Youth Innovation Program”.

## Data availability

The data and materials used in this study are available upon request from the corresponding author. Access to the data and materials may be subject to restrictions based on participant confidentiality and proprietary information. Access requests will be reviewed on a case-by-case basis.

## Figures and Tables

**Figure S1:**
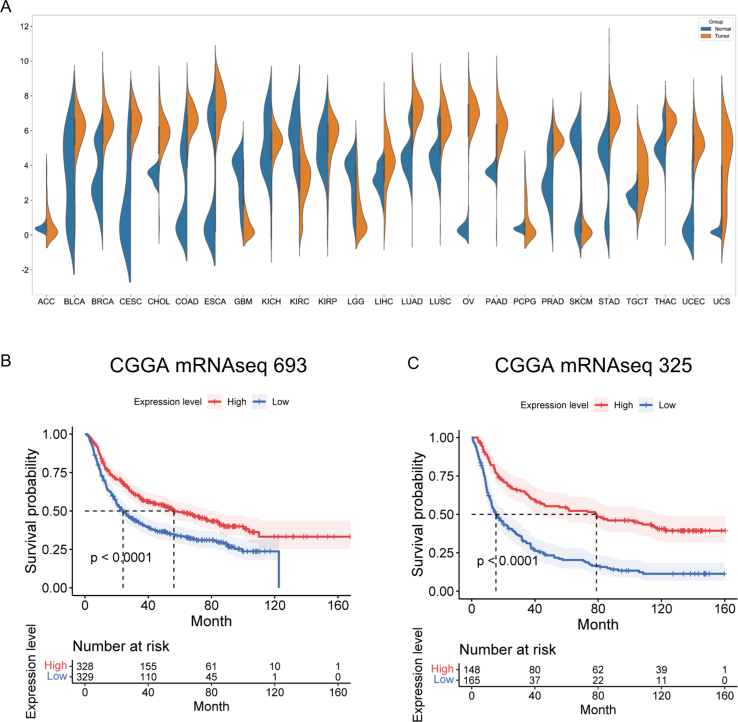


**Figure S2:**
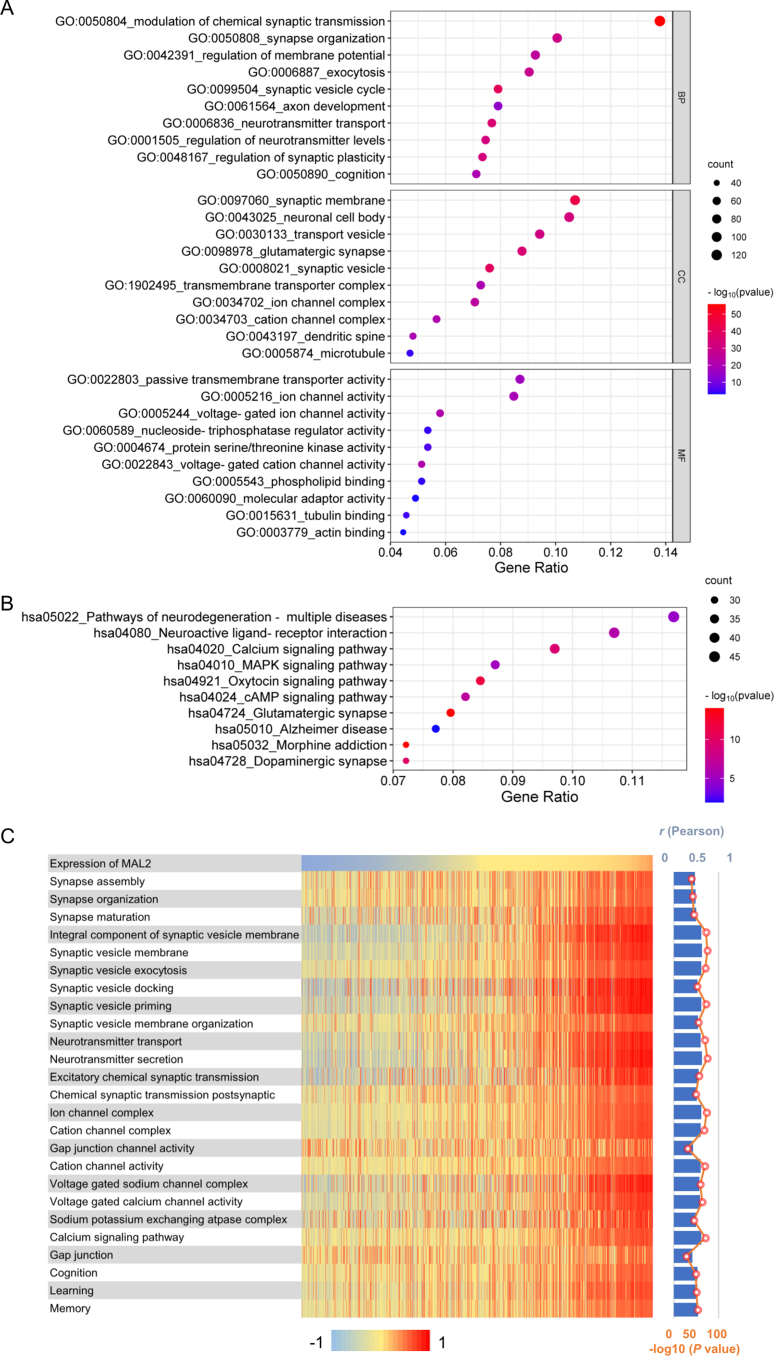


**Figure S3:**
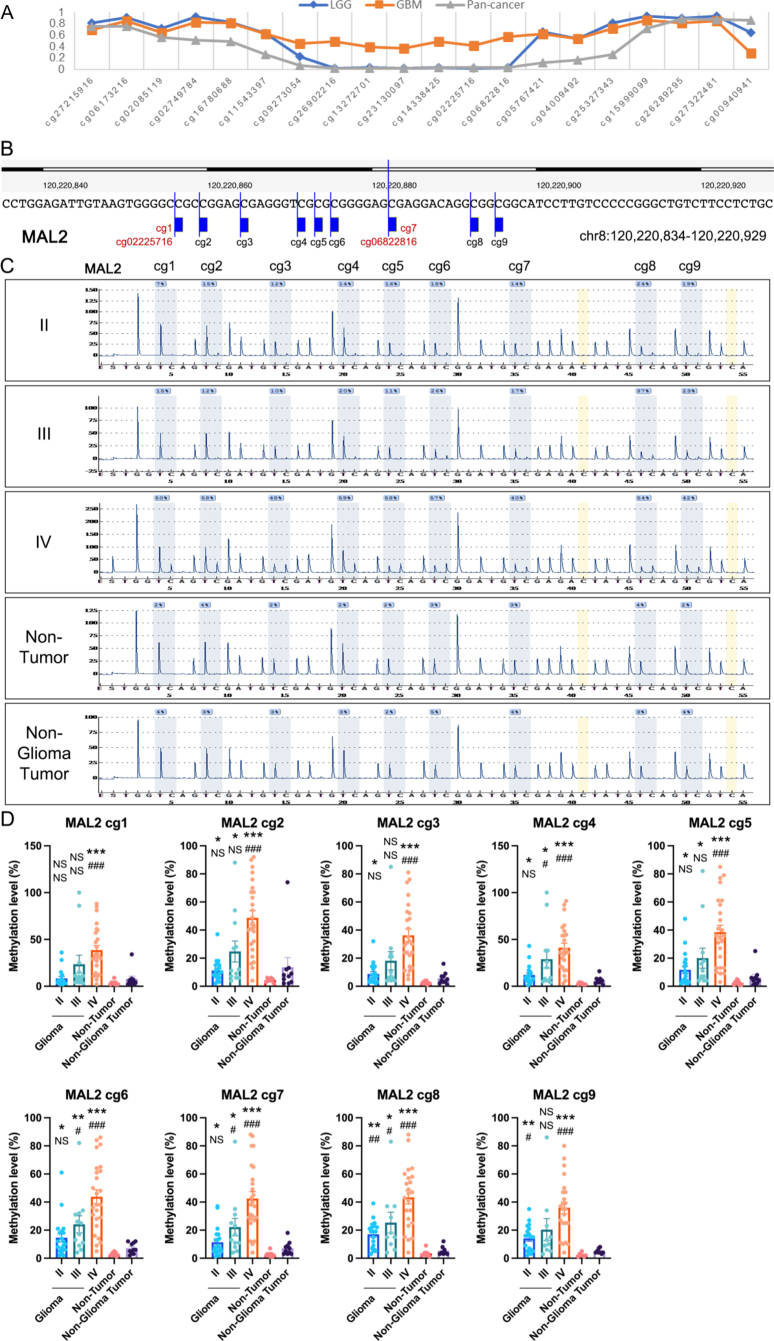


**Figure S4:**
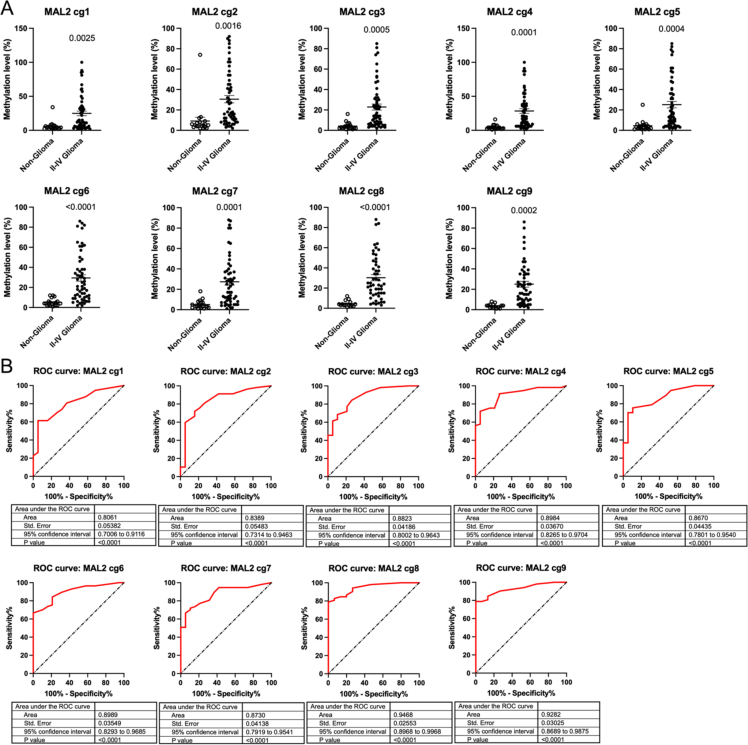


**Figure S5:**
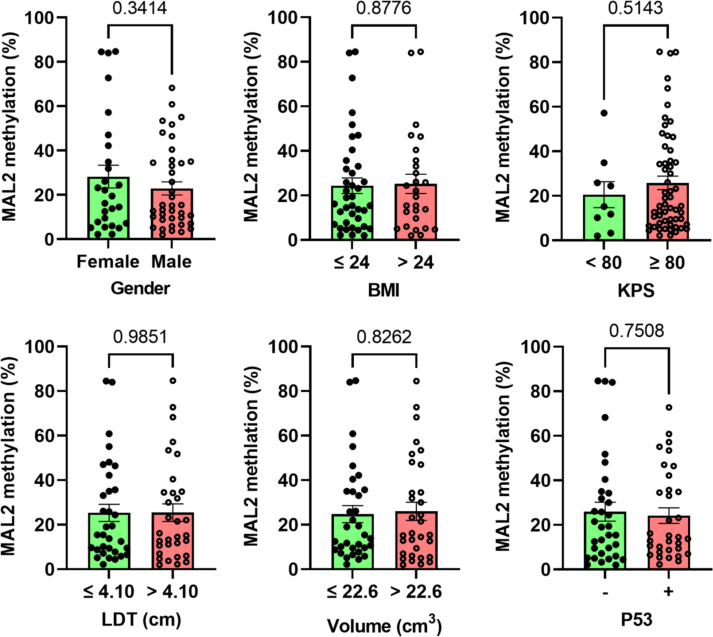


**Figure S6:**