# Supplementary material for: MAL2 DNA methylation serves as a biomarker for the diagnosis and prognosis of glioma
Source: Genes Dis. 2023 Sep 7;11(5):101082. doi: 10.1016/j.gendis.2023.101082 (PMC11145215; doi:10.1016/j.gendis.2023.101082)
Supplement: Multimedia component 1 [file mmc1.docx]

**Material and Methods**

**Patients and samples**

Tissue samples were collected from 84 patients, including 66 glioma patients, 9 non-glioma brain tumor patients and 9 patients with non-tumor brain diseases, who underwent neurosurgical resection at Huashan Hospital, Fudan University from 2020 to 2022（Table S1）. All the samples were pathologically confirmed and immedisately frozen in the -80°C deep freezer. After the tissue samples were pyrosequenced, the glioma patients were also selected for follow-up, and consequently 44 glioma patients in total were analyzed for the prognosis of glioma patients (Table S2). KPS score accesses a patient’s ability to survive chemotherapy based on their state. Additionally, patients always fail to care for themselves if KPS < 80. The information in respect of LDT was obtained from medical imaging. The volume of gliomas was computed from digital caliper raw data by using the formula: Volume (cm^3^) = (l×w^2^)/2. The value of l (length) referred to the longer axis of the maximum cross-section of glioma, and the shorter axis perpendicular to that is the value of w (width). All sorts of pathological characteristics, such as isocitrate dehydrogenase (IDH) mutation, P53, Ki67, CD34, CIC were performed at the Molecular Pathology Testing Center of Huashan Hospital. The collection of samples and clinical information in this study was approved by the Ethics Committee of Huashan Hospital, and our study was approved by The Institutional Review Board Huashan Hospital, Fudan University. Furthermore, written informed consent was achieved from each patient.

**Extraction and bisulfite conversion of tissue genomic DNA**

The whole process of extraction and bisulfite conversion of tissue genomic DNA (gDNA) was executed carefully by manufacturer’s instructions. About 100 mg brain samples of patients was transferred to a 2 ml tube (Eppendorf, Germany) and the gDNA was extracted by the DNA extraction kit (QIAGEN, Germany). The concentration of extracted DNA was measured by NanoDrop Spectrophotometers (Thermo Fisher, MA, USA). Next, about 500 ng extracted gDNA was bisulfite converted with QIAGEN EpiTect Bisulfite Kit, in which the unmethylated cytosines were converted to uracils, while the methylated cytosines kept unchanged. Correspondingly, the unmethylated C would become T, whereas the methylated C were still C after PCR amplification.

**Primer design, PyroMark PCR amplification and Pyrosequencing**

The bisulfite-converted tissue gDNAs were used as template DNAs. The genomic sequence of identified MAL2 CpG site was searched online, and chosen for primer design by using QIAGEN PyroMark Assay Design software. PCR amplification primer F, biotin-tagged R (R-biotin), and pyrosequencing primer S were designed as follows: MAL2 Primer F: 5′-GTTTGTTTGGAGATTGTAAG-3′; MAL2 Primer R-Biotin: 5′-CCTCCCCAAACAATTTC-3′; MAL2 Primer S: 5′-TGTTTGGAGATTGTAAGT-3′.

Referring to the instructions, PCR amplification of bisulfite-converted DNA templates was executed using PyroMark PCR Kit (QIAGEN, 978705). PCR amplification was performed in ThermoFisher Veriti 96-Well thermo cycler, with cycle number between 45-50. The pyrosequencing of MAL2 CpG sites would then be followed by the amplification of bisulfite-converted template DNA by the PyroMark PCR.

According to the manufacturer’s instructions for pyrosequencing, treatment of biotin-labeled PCR products included following: coupled to beads, captured by suction pump, denaturized in NaOH, washed in buffer, and annealed with sequencing primers. Then, processed PCR products were sequenced by using the QIAGEN PyroMark Q24 instrument. In each assay, a non-CpG cytosine was selected as a control for complete bisulfite conversion. The generated original data (pyro.run) were imported in and read by the software QIAGEN PyroMark Q24 2.0.8, then shown as the pyrogram form, and analyzed by Graphpad Prism.

**Data mining and publicly databases**

Firstly, we downloaded MAL2 mRNA expression FPKM in pan-cancers of the TCGA database and in corresponding normal tissues from the GTEx database from an exploration tool called UCSC Xena,^16^ accessible at http://xena.ucsc.edu/, through which varied MAL2 CpG site methylation and clinical data of glioma patients were attained. The genomic sequence of MAL2 was acquired from EWAS Data Hub, which was available at https://ngdc.cncb.ac.cn/ewas/datahub. Through normalizing the expression profile, the integrated data described the MAL2 expression distribution and obtain the differential expression of MAL2 in glioma and normal brain tissues. The MAL2 expression TPM was obtained and complied from the TCGA database^17, 18^ (https://www.cancer.gov/tcga) to analyze the correlation with clinical characters and survival prognosis. Next mRNAseq_693 dataset of CGGA database^19^ was sourced to confirm the correlation between the expression of MAL2 and disparate clinicopathological characteristics (http://www.cgga.org.cn/).

**GO, KEGG and GSVA analysis of MAL2**

MAL2-related genes were selected by Pearson correlation analysis (*r* > 0.5, *P* < 0.05), and the genes would be counted out provided the average gene expression were less than 0.5. The Database for Annotation, Visualization, and Integrated Discovery (DAVID) was used to analyze the selected genes. Selecting official gene symbol and Homo sapiens as species, we obtained GO and KEGG enrichment analysis results. The gene list of MAL2-related biological functions was achieved from AmiGO2 portal (http://amigo.geneontology.org/amigo). Under default parameters, the functional enrichment score was calculated by the given package (R environment).

**Statistical Analysis**

Statistical analyses and visualization were performed using SPSS software (version 22.0 IBM), GraphPad Prism software (version 9.0), and R (version 4.0.0). And box plots and violin plots were described by https://www.bioinformatics.com.cn/, an online platform for data analysis and visualization. The difference between the two groups was assessed by unpaired *t* test. The significance of the correlation between the two groups was tested by Pearson correlation analysis. The high and low expression of DNA groups and the high and low methylation level groups were divided according to the appropriate intermediate values of data. Multivariable logistic regression was used to construct the prediction model, and the variance inflation factor of these CpG sites were all less than 5. ROC curves were used to evaluate the prediction performance of the model. KM curves evaluated the prognostic value. Univariate Cox regression assessed the significance of the prognostic value, CpG sites with *P* < 0.05 was chosen for LASSO Cox regression with the R package, glmnet, and the constructed model was used for signature construction. The clinical parameters were evaluated by univariate Cox regression and variables would be included in the multivariate Cox regression, provided *P* < 0.2. The results were considered relevant and statistically significant when *P* < 0.05.

**Figure S1.** MAL2 expression profiling and KM curves of MAL2 expression in glioma of CGGA database.

**(A)** MAL2 expression profiling in 25 different common cancers and corresponding normal tissues. **(B)** Patients with high expression of MAL2 have longer median overall survival in the CGGA mRNAseq 693 dataset. **(C)** At the same cutoff value, patients with high expression of MAL2 have longer median overall survival in the CGGA mRNAseq 325 dataset.

**Figure S2.** The relevant biological functions with MAL2 through GO and KEGG enrichment analysis and GSVA**.**

(**A**) For GO enrichment analysis, the biological process (BP), cellular component (CC), molecular function (MF) mostly related to MAL2 in the TCGA database. (**B**) KEGG pathway enrichment analysis of MAL2 in the TCGA database. (**C**) The heatmap showed MAL2 expression and the enrichment scores of its mainly correlated biological functions of each sample in the TCGA database.

**Figure S3.** Identification and pyrosequencing of MAL2 candidate CpG sites.

(**A**) Comparing the methylation status at diverse known MAL2 CpG sites of LGG and GBM along with pan-cancer. (**B**) The candidate MAL2 CpG sites of cg02225716, and cg06822186, as well as the unknown MAL2 CpG sites around them, were respectively recorded as cg1 to cg9. (**C**) The selected MAL2 CpG sites were pyro-sequenced in grade II-IV (n = 66), non-tumor brain diseases (n = 9) and non-glioma brain tumor tissue samples (n = 9). (**D**) The methylation level in non-tumor brain diseases and non-glioma brain tumors were far lower than that in glioma, and with the increase of glioma grade, the methylation level gradually increased at cg1 to cg9. (**P* < 0.05, ***P* < 0.01, ****P* < 0.001, II/III/IV Glioma vs. Non-Tumor. #*P* < 0.05, ##*P* < 0.01, ###*P* < 0.001, II/III/IV Glioma vs. Non-Glioma Tumor).

**Figure S4.** The construction of MAL2 methylation-based diagnostic model.

(**A**) There were significant differences in glioma and non-glioma at MAL2 CpG sites of cg1 to cg9. (**B**) There was a relatively large area under the ROC curve of MAL2 cg1 (cg02225716) (AUC = 0.8061, 95% CI: 0.7006-0.9116, *P* < 0.0001). MAL2 cg2 AUC = 0.8389, 95% CI: 0.7314-0.9563, *P* < 0.0001. MAL2 cg3 AUC = 0.8823, 95% CI: 0.8002-0.9643, *P* < 0.0001. MAL2 cg4 AUC = 0.8984, 95% CI: 0.8264-0.9704, *P* < 0.0001. MAL2 cg5 AUC = 0.8670, 95% CI: 0.7801-0.9540, *P* < 0.0001. MAL2 cg6 AUC = 0.8989, 95% CI: 0.8293-0.9685, *P* < 0.0001. MAL2 cg7 (cg06822816) AUC = 0.8730, 95% CI: 0.7919-0.9541, *P* < 0.0001. ROC curves of MAL2 cg8 and cg9 presented extremely large areas under the curve. MAL2 cg8 AUC = 0.9468, 95% CI: 0.8968-0.9968, *P* < 0.001. MAL2 cg9 AUC = 0.9282, 95% CI: 0.8689-0.9875, *P* < 0.001.

**Figure S5.** The correlation of MAL2 methylation with clinical characteristics in glioma.

There was no difference of MAL2 average methylation level in different genders, BMI groups, KPS groups, longest-diameter of tumor, volume, P53 groups.

**Figure S6.** Methylation of MAL2 displayed significant prognostic value in glioma.

**(A)** Through KM analysis, at MAL2 cg1 (cg02225716) and cg2, patients with lower methylation level had longer survival (P < 0.05). At MAL2 cg3 to cg9, although the difference of survival in different methylation level group is not significant, the trend is similar. (B) Risk plot showed with the increase of risk, OS was shortened and the deaths increased. (C) PCA plot showed patients with high and low risk could be well divided.

**Table S1** **Tissue samples and clinical characteristics of various brain diseases**

|  | Glioma (n = 66) | |  | Other tumors (n = 9) | |  | Non-tumor (n = 9) | |
| --- | --- | --- | --- | --- | --- | --- | --- | --- |
|  | n | (%) |  | n | (%) |  | n | (%) |
| **Age** |  | |  |  | |  |  | |
| ≤ 50 | 28 (42.4) | |  | 3 (33.3) | |  | 7 (77.8) | |
| > 50 | 38 (57.6) | |  | 4 (44.4) | |  | 0 (0.0) | |
| NA | 0 (0.0) | |  | 2 (22.2) | |  | 2 (22.2) | |
| **Gender** |  | |  |  | |  |  | |
| Male | 39 (59.1) | |  | 5 (55.6) | |  | 5 (55.6) | |
| Female | 27 (40.9) | |  | 2 (22.2) | |  | 2 (22.2) | |
| NA | 0 | |  | 2 (22.2) | |  | 2 (22.2) | |
| **BMI** |  | |  |  | |  |  | |
| ≤ 24 | 39 (59.1) | |  | 5 (55.6) | |  | 5 (55.6) | |
| > 24 | 25 (37.9) | |  | 2 (22.2) | |  | 2 (22.2) | |
| NA | 2 (3.0) | |  | 2 (22.2) | |  | 2 (22.2) | |
| **KPS score** |  | |  |  | |  |  | |
| ≥ 80 | 57 (86.4) | |  | / | |  | / | |
| < 80 | 9 (13.6) | |  | / | |  | / | |
| **LDT* (cm)** |  | |  |  | |  |  | |
| ≤ 4.3 | 34 (51.5) | |  | / | |  | / | |
| > 4.3 | 31 (47.0) | |  | / | |  | / | |
| NA | 1 (1.5) | |  | / | |  | / | |
| **Tumor volume (cm^3^)** |  | |  |  | |  |  | |
| ≤ 30.8 | 41 (62.1) | |  | / | |  | / | |
| > 30.8 | 24 (36.3) | |  | / | |  | / | |
| NA | 1 (1.5) | |  | / | |  | / | |
| **Grade** |  | |  |  | |  |  | |
| II | 22 (33.3) | |  | / | |  | / | |
| III | 14 (21.2) | |  | / | |  | / | |
| IV | 30 (45.5) | |  | / | |  | / | |
| **IDH status** |  | |  |  | |  |  | |
| Mutant | 27 (40.9) | |  | / | |  | / | |
| Wildtype | 39 (59.1) | |  | / | |  | / | |
| **P53** |  | |  |  | |  | / | |
| Positive | 33 (50.0) | |  | / | |  | / | |
| Negative | 33 (50.0) | |  | / | |  | / | |
| **Ki67 (%)** |  | |  |  | |  |  | |
| ≤ 10 | 37 (56.1) | |  | / | |  | / | |
| > 10 | 29 (43.9) | |  | / | |  | / | |
| **ATRX** |  | |  |  | |  |  | |
| Expressed | 46 (69.7) | |  | / | |  | / | |
| Lost | 20 (30.3) | |  | / | |  | / | |
| **BRAFV600E** |  | |  |  | |  |  | |
| Positive | 9 (13.6) | |  | / | |  | / | |
| Negative | 57 (86.4) | |  | / | |  | / | |
| **CD34** |  | |  |  | |  |  | |
| Positive | 39 (59.1) | |  | / | |  | / | |
| Negative | 27 (40.9) | |  | / | |  | / | |
| **H3K27ME3** |  | |  |  | |  |  | |
| Positive | 31 (47.0) | |  | / | |  | / | |
| Negative | 35 (53.0) | |  | / | |  | / | |
| **FuBP1** |  | |  |  | |  |  | |
| Positive | 52 (78.8) | |  | / | |  | / | |
| Negative | 14 (21.2) | |  | / | |  | / | |
| **CIC** |  | |  |  | |  |  | |
| Positive | 45 (68.2) | |  | / | |  | / | |
| Negative | 21 (31.8) | |  | / | |  | / | |

*LDT: Longest diameter of tumor

| **Table S2 Clinical characteristics of follow-up patients** | |
| --- | --- |
| Characteristics | n (%) |
| **Age** |  |
| ≤ 50y | 20 (45.5) |
| > 50y | 24 (54.5) |
| **Gender** |  |
| Female | 19 (43.2) |
| Male | 25 (56.8) |
| **BMI*** |  |
| ≤ 24.0 | 24 (54.5) |
| > 24.0 | 18 (40.9) |
| **Longest diameter of tumor (cm)** | |
| ≤ 4.4 | 24 (54.5) |
| > 4.4 | 20 (45.5) |
| **Tumor volume (cm^3^)** |  |
| ≤ 31.0 | 30 (68.2) |
| > 31.0 | 14 (31.8) |
| **WHO category** |  |
| II | 17 (38.6) |
| III | 10 (22.8) |
| IV | 17 (38.6) |
| **KPS score**** |  |
| ≤ 80 | 6 (13.6) |
| > 80 | 38 (86.4) |
| **P53** |  |
| Negative | 23 (52.3) |
| Positive | 21 (47.7) |
| **IDH mutant status** |  |
| Wildtype | 25 (56.8) |
| Mutant | 19 (43.2) |
| **Ki67 (%)** |  |
| ≤ 10 | 27 (61.4) |
| > 10 | 17 (38.6) |
| **Tumor resection** |  |
| Total or nearly total | 37 (84.1) |
| Partial or biopsy | 7 (15.9) |
| **Treatment** |  |
| None | 1 (2.3) |
| Chemotherapy only | 2 (4.5) |
| Radiotherapy only | 3 (6.8) |
| Chemotherapy and Radiotherapy | 38 (86.4) |

*Two of the patients’ BMI data are missing.

** KPS score: Karnofsky Performance Scale status score
